# Physical and Chemical Attributes and Yield of Sour Passion Fruit Grown under Deficit Irrigation Strategies and Salicylic Acid Application

**DOI:** 10.1021/acsomega.6c04904

**Published:** 2026-09-23

**Authors:** Allesson R de Souza, Geovani S. de Lima, André A. R Da Silva, Carlos Alberto V de Azevedo, Lucyelly Dâmela A Borborema, Kheila G Nunes, Denis S Costa, Larissa Fernanda S. Santos, Brencarla de M Lima, Valeska Karolini N Oliveira, Smyth T. de A. Silva, Vitor Manoel B da Silva, Edilene D de Araújo, Allysson Jonhnny T. Mendonça, Lauriane A. dos A. Soares, Hans R Gheyi

**Affiliations:** † Academic Unit of Agricultural Engineering, Federal University of Campina Grande, Campina Grande 58429-900, Brazil; ‡ Academic Unit of Agrarian Sciences, Federal University of Campina Grande, Pombal 58840-000, Brazil; § Academic Unit of Agronomy, 674633 Universidade Federal do Oeste do Pará, Juruti 68170-000, Brazil; ∥ Food Technology Department, State University of Campinas, Campinas 13083-862, SP, Brazil; ⊥ Postgraduate Program in Engineering and Management of Natural Resources, Federal University of Campina Grande, Campina Grande 58430-380, Brazil

## Abstract

With population growth, increasing agricultural productivity is essential, particularly in semiarid regions, regions experiencing drought problems. Thus, strategies aimed at mitigating the effects of water deficit and increasing water use efficiency become essential for the sustainability of agricultural production. In this context, the objective of present study was to evaluate the effect of salicylic acid application on the attenuation of water deficit on the physical and chemical attributes and yield components of sour passion fruit at different phenological stages. The treatments were arranged in a randomized block design, in a 6 × 2 factorial scheme, consisting of six deficit irrigation strategies based on reference evapotranspiration of crop combined with the presence and absence of salicylic acid (SAL) application, with four replications and one plant per plot in each drainage lysimeter. It was observed that the deleterious effects of water deficit under irrigation with 50% ETr during the flowering stage were mitigated by the application of 1.0 mM salicylic acid, promoting increases in yield–related variables. The application of 1.0 mM salicylic acid increased yield and water use efficiency of sour passion fruit plants under flowering stage. In addition, salicylic acid application increased pH, vitamin C, soluble solids, titratable acidity, the soluble solids to titratable acidity ratio, reducing sugars, flavonoids, anthocyanins, and phenolic compounds.

## Graphical abstract

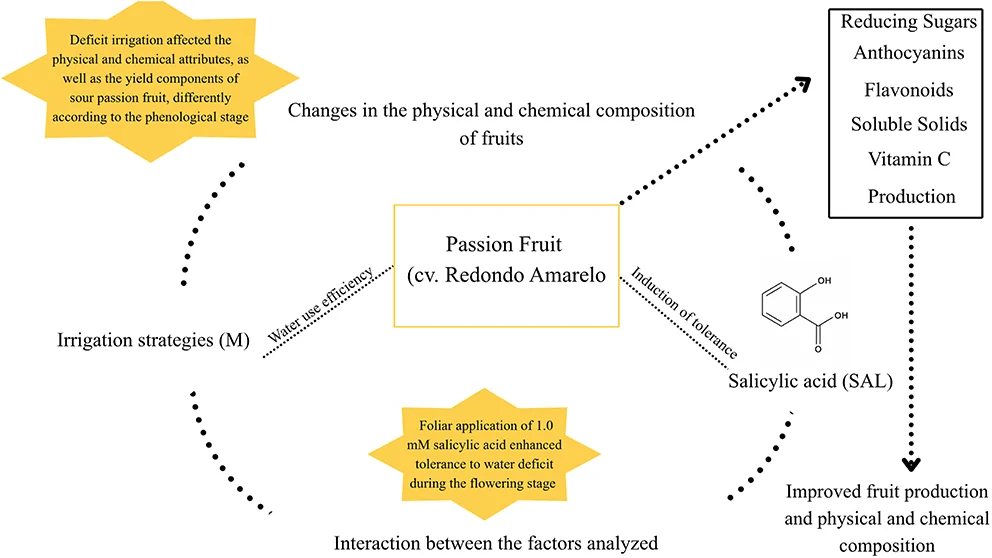

## 1 Introduction

The globalization process, combined with the growing population demand, has greatly increased the global demand for food.^1^ However, changes in climate patterns have significantly affected meteorological conditions, resulting in losses to the agricultural sector.^2^ Among the main consequences of these changes, drought stands out as one of the most severe abiotic constraints on crop production in semiarid regions of Northeastern Brazil.^3^

Water scarcity induces partial stomatal closure, which reduces carbon fixation in plants, leading to lower CO_2_ assimilation and resulting in excess light energy that is not used in the photochemical phase of photosynthesis.^4^ When this excess energy is transferred to O_2_ in the thylakoids, it may lead to the formation of reactive oxygen species (ROS), causing a series of physiological disturbances, photo–oxidative damage, and restrictions on photosynthetic efficiency.^5,6^

The irrigation water depth has an important impact on plant biomass accumulation and nutrient transport, directly affecting fruit yield and quality.^7^ In this context, deficit irrigation with variation across phenological stages emerges as a promising strategy for improving water use efficiency.^8^ Deficit irrigation levels have been applied to plants throughout the entire vegetative stage or during specific developmental stages, which may lead to improvements in fruit quality without compromising yield.^9^

It is also important to consider the induction of plant tolerance to water deficit through foliar application of salicylic acid (SAL), which is characterized as a promising strategy for cultivation in areas under water scarcity conditions.^10^ Salicylic acid is a phenolic phytohormone that can act in inducing plant tolerance to abiotic stresses by modulating metabolic responses associated with the accumulation of proline, proteins, secondary metabolites, and sugars, thereby contributing to more efficient osmotic adjustment.^11,12^ In addition, the beneficial effect of salicylic acid has been attributed to its role in activating the antioxidant system, with increased activity of antioxidant enzymes and protection of cell membranes against oxidative damage.^13^

Studies have been conducted evaluating the effects of salicylic acid (SAL) on the mitigation of abiotic stresses, such as the work by Sobrinho et al.^14^ with sour passion fruit under salinity stress, in which the authors observed that application at concentrations ranging from 1.0 to 1.4 mM SAL promoted increases in yield and fruit physical attributes. However, studies addressing sour passion fruit under water stress conditions, considering variation across phenological stages combined with salicylic acid application, remain incipient in the literature.

Brazil is recognized as the world’s largest producer and exporter of passion fruit.^15^ The Northeastern region of the country stands out as the main producing area, accounting for 69.77% of the 607,859 tons produced nationwide in 2022. However, the average yield in this region is only 14.76 t ha^–1^, which is lower than that of the Southern region of Brazil, where yields reach 21.29 t ha^–1^.^16^ This difference may be associated with the edaphoclimatic characteristics of the region. The Northeast region of Brazil is characterized by irregular rainfall and high evaporation rates during most months of the year, which limits water availability for crops and results in lower productivity.^17^

This research was based on the hypothesis that the effects of deficit irrigation distinctly influence the yield and postharvest quality of sour passion fruit, and that foliar application of 1.0 mM salicylic acid may induce tolerance to water deficit by increasing enzymatic activity and reducing damage to the photosynthetic apparatus, which may result in greater translocation of photoassimilates to the fruits. Therefore, the objective of this study was to evaluate the effect of salicylic acid application on the attenuation of water deficit on the yield and postharvest quality of sour passion fruit at different phenological stages.

## 2 Materials and Methods

### 2.1 Experimental Site

The study was carried out between December 2023 and August 2024 in a greenhouse at the Academic Unit of Agricultural Engineering, Federal University of Campina Grande, Campina Grande, Paraíba, Brazil (07°15′18″ S, 35°52′28″ W; 550 m above sea level). The experiment was performed in an arch-shaped greenhouse measuring 30 m in length, 21 m in width, and 3.0 m in height, covered with a 150-μm low-density polyethylene film. The meteorological data collected during the experimental period are presented in Figure 1.

**Figure 1:**
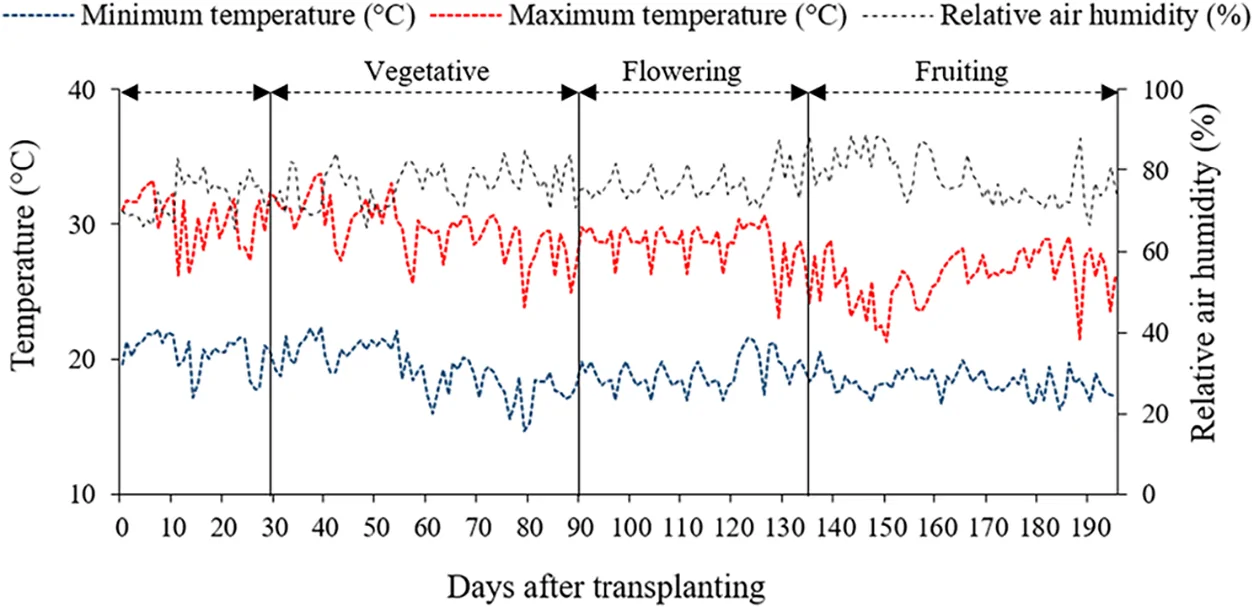
Maximum and minimum temperature and relative air humidity of the greenhouse internal environment during the period from December 27, 2023, to August 04, 2024.

### 2.2 Statistical Design and Treatments

The study was arranged in a randomized block design using a 6 × 2 factorial scheme. The treatments comprised six irrigation management strategies established according to crop reference evapotranspiration (ETr): irrigation with 100% ETr throughout the entire cultivation cycle—M1 (30–196 days after transplanting—DAT); and irrigation with 50% ETr during the following phenological stages: vegetative stage—M2 (30–90 DAT); flowering stage—M3 (90–136 DAT); fruiting stage—M4 (136–196 DAT); successive vegetative/flowering stages—M5 (30–136 DAT); and vegetative/fruiting stages—M6 (30– 90/136–196). These strategies were evaluated in the presence or absence of salicylic acid—SAL application, being 0 and 1 mM, using four replications and one plant per plot in each drainage lysimeter. The SAL concentration was selected based on the study by Galvão Sobrinho et al.,^14^ who investigated foliar SAL applications in sour passion fruit plants exposed to saline irrigation water with electrical conductivity ranging from 0.8 to 4.0 dS m^–1^.

### 2.3 Experiment Conduction and Treatment Application

Sour passion fruit seedlings (cv. Redondo Amarelo) were produced by sowing three seeds per 15 × 20 cm^2^ plastic bag (3 kg capacity) containing a substrate composed of soil, sand, and earthworm humus (84:15:1, on a volume basis). After seedling emergence, thinning was carried out to retain the most vigorous plant in each container. The remaining seedlings were then supported with stakes to promote vertical growth and minimize lodging.

At 80 days after sowing (DAS), the seedlings were transplanted into plastic pots with a volume of 200 dm^3^, adapted as drainage lysimeters, using transparent drains with a diameter of 20 mm. In each lysimeter, a geotextile fabric (Bidim type) was placed over a 1 kg layer of gravel (No. 0) to prevent drain obstruction. Beneath each unit, two plastic bottles with a capacity of 2 L were attached to collect drained water, allowing the estimation of plant water consumption.

Afterward, each pot received 250 kg of Regolithic Neosol obtained from the 0–30 cm soil layer. The soil’s physical and chemical properties were assessed following the methodology proposed by Teixeira et al.:^18^ pH = 5.40; organic matter = 17.42 g dm^–3^; phosphorus (P) = 2.92 mg dm^–3^. The exchangeable cation contents were potassium (K) at 0.28 cmol_c_ kg^–1^, sodium (Na) at 0.04 cmol_c_ kg^–1^, calcium (Ca^2+^) at 1.87 cmol_c_ kg^–1^, and magnesium (Mg^2+^) at 1.70 cmol_c_ kg^–1^. Elements related to soil acidity included aluminum (Al^3+^). Regarding physical attributes, particle size analysis showed the following composition: 675.2 g kg^–1^ of sand, 221.8 g kg^–1^ of silt, and 103 g kg^–1^ of clay. Soil bulk density was 1.51 g cm^–3^, and soil moisture content was 5.34 dag kg^–1^.

The irrigation treatments were applied daily at 7:00 a.m. The volume supplied to each container was calculated using the soil water balance, with deficit irrigation corresponding to 50% of crop reference evapotranspiration (ETr) imposed according to the respective phenological stage. Soil samples were collected from the 0–20 and 20–40 cm layers at each transition between developmental stages to determine soil water content using the gravimetric method.^19^

Phenological stages were identified according to plant morphological development. The vegetative stage extended from 30 to 90 DAT, encompassing the period from transplanting until the formation of the floral primordium. Flowering occurred between 90 and 136 DAT and was considered to begin with the appearance of the floral primordium and end at anthesis. The fruiting stage lasted from 136 to 196 DAT, starting with fruit set and extending until the beginning of partial fruit yellowing. Stage transitions were determined following the criteria proposed by Pinheiro et al.,^17^ with the subsequent phenological phase considered established when at least 50% of the plants displayed the characteristic features of that stage.

Water consumption for each irrigation strategy is presented in Table 1S. Deficit irrigation management strategies began at 30 DAT, when the plants reached a height of 130 cm.

Salicylic acid solutions were freshly prepared before each application by first dissolving the compound in 30% ethyl alcohol to overcome its limited solubility in water at room temperature. The surfactant Wil fix was then incorporated at a concentration of 0.5 mL L^–1^ to improve droplet spreading and adhesion on the leaf surface. Foliar applications were initiated at 20 DAT and repeated every 15 days by spraying both adaxial and abaxial leaf surfaces between 5:00 and 6:00 p.m., using a Jacto backpack sprayer, model Jacto XP, with a capacity of 12 L, a maximum working pressure of 88 psi (6 bar), and a JD 12P nozzle. In plants not treated with salicylic acid, a solution composed of distilled water, ethyl alcohol, and an adhesive adjuvant (Wil fix) was applied. In total, five applications per plant were carried out until the flowering stage, resulting in a total spray volume of 2.2 L per plant. To prevent salicylic acid drift during application, a plastic curtain was used to cover the neighboring plants, thereby avoiding unintended application to other treatments.

Nutrient management followed the recommendations of da Costa et al.^20^ Phosphorus was supplied as a basal application at a rate of 120 g per plant using single superphosphate (18% P_2_O_5_, 16% Ca^2+^, and 10% S). Nitrogen and potassium were provided through biweekly fertigation starting at 15 DAT, with urea (45% N) and potassium chloride (60% K_2_O) serving as the respective nutrient sources. During the vegetative and flowering stages, plants received 166.57 g of N and 156.58 g of K_2_O per plant, whereas 140.00 g of N and 360.00 g of K_2_O per plant were applied during fruit development. Micronutrients were supplied throughout the crop cycle by foliar spraying at 15-day intervals using a backpack sprayer and a solution containing 1.0 g L^–1^ of Dripsol micro (1.2% Mg, 0.85% B, 3.4% Fe, 4.2% Zn, 3.2% Mn, 0.5% Cu, and 0.06% Mo).

Plants were trained using a vertical trellis system supported by the greenhouse structure and No. 14 galvanized wire installed 2.0 m above the soil surface. Each plant was conducted with a single main stem and tied to a 10 mm polypropylene twine until reaching the support wire. After the main stem extended approximately 10 cm beyond the trellis, the apical meristem was removed to induce the development of two secondary branches, which were trained horizontally in opposite directions until they reached a length of 1.0 m. A second pruning of the apical buds was then performed to stimulate the emergence of tertiary branches, which were allowed to grow downward to approximately 30 cm above the soil surface. Throughout the cultivation period, tendrils and lateral shoots were periodically removed to maintain the training system and favor plant growth, following the recommendations of Pinheiro et al.^21^

Flowers were hand-pollinated throughout the flowering period until flower production ended. Crop management practices, including weed removal, soil loosening, and routine phytosanitary maintenance, were performed whenever required during the experimental period. Plants were regularly inspected for the occurrence of pests and diseases, and control measures were implemented as needed. When infestations were detected, insecticide applications containing the active ingredients abamectin or chlorfenapyr were carried out to control aphids (*Aphis gossypii*), mealybugs (*Phenacoccus solenopsis*), whiteflies (*Bemisia tabaci*), passion fruit butterflies (*Dione juno juno*), and southern green stink bugs (*Nezara viridula*).

### 2.4 Variables Analyzed

Harvesting and evaluation of the production components were carried out between 156 and 196 DAT, using fully mature fruits with uniform yellow coloration over the entire peel. For the physical and chemical characterization, 20 fruits were collected from each experimental unit, and their measurements were used to obtain the mean values for the respective variables.

The number of fruits per plant (NFP) was determined by direct counting of all harvested fruits. Average fruit weight (AFW), average peel weight (ASW), and average pulp weight (APW) were estimated by dividing sum of respective weight by number of fruits used in each determination. Pulp yield (PY) was calculated as the ratio between pulp mass and fresh fruit mass. The Production Stability Index (PSI) was determined according to the methodology described by Nautyal et al.,^22^ based on the ratio between yield under treatments and yield of control plants. During the same period, physical characterization of the fruits was performed by measuring equatorial diameter (EDF), polar diameter (PDF), and peel thickness (PT) using a digital caliper.

After harvest, pulp extraction was performed manually and immediately stored in a freezer at −10 °C in hermetically sealed containers. Postharvest quality variables were determined for hydrogen potential (pH), vitamin C content (Vit. C; mg 100 g^–1^ pulp), soluble solids (SS; °Brix), titratable acidity (TA; % citric acid), the ratio between soluble solids and titratable acidity (SS/TA), reducing sugars (RS), flavonoids (Flav.), anthocyanins (Ant.), and phenolic compounds (PC).

The pH was measured by immersing the electrode directly into the diluted sample after calibration of the pH meter with standard buffer solutions (pH 4.0 and 7.0), and the value was recorded once the reading had stabilized.^23^ For vitamin C determination, 1 g of sample was transferred to a 250 mL Erlenmeyer flask containing 50 mL of 0.5% oxalic acid solution. The suspension was homogenized using a Vortex mixer (Warmnest, Salvador, Brazil) and subsequently titrated with 2,6-dichlorophenolindophenol (DCPIP) until the end point, indicated by the appearance of a stable light-pink color, was reached.^24,25^

Soluble solids (SS) were measured with a portable digital refractometer (Atago, PAL-1 model). Titratable acidity (TA) was determined of by Instituto Adolfo Lutz,^26^ and the SS/TA ratio was calculated from the relationship between these two variables. Reducing sugars were quantified by the Anthrone colorimetric method according to Yemn and Willis.^27^ Total flavonoids and anthocyanins were analyzed following the protocol of Francis.^28^ For pigment extraction, 1 g of sample was combined with 10 mL of an extraction solution containing 95% ethanol and 1.5 N HCl (85:15, v/v). After homogenization and maceration for 1 min, the extracts were transferred to aluminum foil-wrapped tubes and stored for 24 h under dark conditions. Absorbance was subsequently recorded with a spectrophotometer at 374 nm for flavonoids and 535 nm for anthocyanins.

An aqueous extract was prepared by grinding 1 g of sample with distilled water in a mortar. The resulting suspension was quantitatively transferred to a 50 mL volumetric flask, completed to volume with distilled water, and maintained at rest for 30 min. After filtration, 900 μL of the extract was combined with 1225 μL of distilled water and 125 μL of Folin–Ciocalteu reagent for determination of total phenolic compounds.

Fruit yield per plant was determined by weighing the fruits at harvest, which occurred between 136 and 196 DAS, when the peel had reached complete yellow coloration. Water use efficiency was obtained from the ratio of yield to water consumption under each irrigation strategy, with the corresponding values provided in Table 1S.

Initially, the data were assessed for normality using the Shapiro–Wilk test and for homogeneity of variances using Levene’s test. Mean comparisons among deficit irrigation strategies were performed using the Scott–Knott test (*p* ≤ 0.05), while Tukey’s test (*p* ≤ 0.05) was used for the salicylic acid treatments. Pearson’s correlation analysis was subsequently conducted among all evaluated variables at *p* ≤ 0.05.

The original data set was subjected to Principal Component Analysis (PCA) to reduce data dimensionality and summarize the variability of the original variables into a smaller number of orthogonal components while preserving most of the information contained in the data set. Principal components were retained based on eigenvalues (λ ≥ 1.0) obtained from the correlation matrix and on components that individually explained more than 10% of the total variance. Within each component, only variables with absolute factor loadings greater than |0.60| were considered, following Souza et al.,^29^ as these variables contributed most strongly to the interpretation of the principal axes.

Subsequently, orthogonal Varimax rotation was applied to maximize the variance of the factor loadings and facilitate the interpretation of the principal components while preserving their independence. Based on the variables retained after rotation, *k*-means clustering was performed to classify the treatments into groups with similar characteristics, thereby identifying response patterns among the evaluated variables. All statistical analyses were performed using RStudio software (version 4.1.0), with the aid of the *AgroR*, *ggcorrplot*, *mclust* and *metan* packages.

## 3 Results

A significant interaction between irrigation strategies (M) and salicylic acid application (SAL) was detected for all evaluated variables (Table 2S).

The number of fruits per plant (Figure 2a) of sour passion fruit cultivated without salicylic acid application was higher under strategies M1, M2, M4, M5, and M6 compared to plants irrigated with 50% ETr during the flowering stage (M3). When 1.0 mM salicylic acid was applied, the NFP of plants cultivated under water deficit during the vegetative/fruiting stages (M6) differed significantly from those subjected to strategies M1, M2, M3, M4, and M5. Analyzing the effect of salicylic acid application within each irrigation strategy, it was observed that the application of 1.0 mM SAL increased NFP in plants irrigated under water deficit during in M3, whereas a reduction was observed in M6.

**Figure 2:**
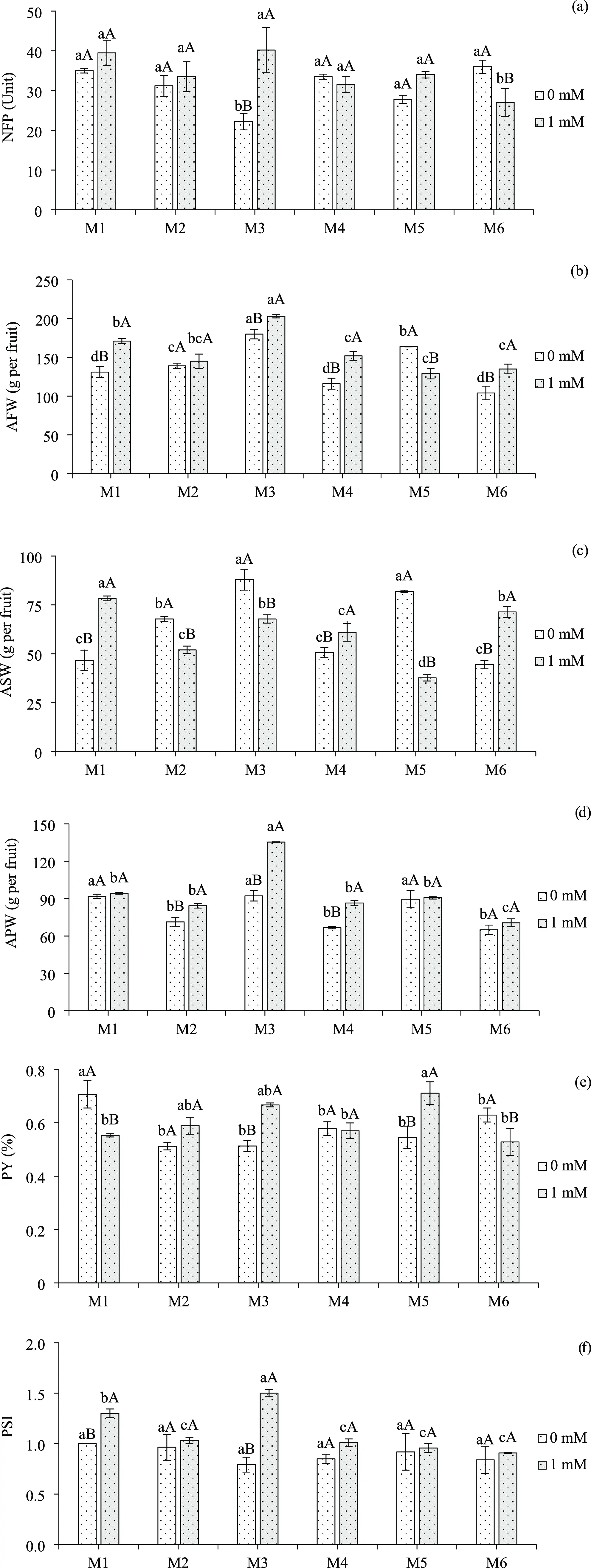
Number of fruits per plant—NFP (a), average fruit weight—AFW (b), peel weight—ASW (c), pulp weight—APW (d), pulp yield—PY (e), and production stability index—PSI (f) of sour passion fruit cv. Redondo Amarelo as a function of the interaction between deficit irrigation strategies and salicylic acid application. Lowercase letters denote the absence of significant differences among deficit irrigation strategies according to the Scott–Knott test (*p* ≤ 0.05), whereas uppercase letters indicate the absence of significant differences between salicylic acid treatments based on Tukey’s test (*p* ≤ 0.05). Vertical bars correspond to the standard error of the mean (*n* = 4).

For average fruit weight (Figure 2b), plants cultivated with 0 mM SAL under deficit irrigation during the flowering stage (M3) exhibited the highest value (180 g per fruit), representing an increase of 37.40% compared to treatment M1 (131 g per fruit). However, application of 1.0 mM SAL resulted in higher AFW in plants subjected to M1, M3, M4, and M6 compared to those cultivated under water deficit during the vegetative/flowering stages (M5). When comparing the effects of salicylic acid within each irrigation strategy (Figure 2b), application of this phytohormone led to statistically higher AFW in plants cultivated under M1, M3, M4, and M6 relative to those that did not receive SAL. In plants subjected to water deficit during M5, application of 1.0 mM SAL resulted in lower AFW compared to plants grown under 0 mM. Conversely, no significant differences in AFW were observed between salicylic acid application in plants subjected to irrigation strategy M2.

Average peel weight (Figure 2c) of plants cultivated without salicylic acid application and subjected to water deficit during M3 and M5 differed significantly from those under M1, M2, M4, and M6. In plants that received foliar application of salicylic acid, the highest ASW value (78.3 g per plant) was observed under full irrigation (100% ETr) throughout the entire crop cycle (M1), being superior compared to the other irrigation strategies (M2, M3, M4, M5, and M6). When analyzing the effect of the tested SAL concentrations within each deficit irrigation strategy, application of 1.0 mM SAL reduced ASW in plants cultivated under M2, M3, and M5, while increasing average peel weight in treatments M1, M4, and M6.

Pulp weight (Figure 2d) of plants cultivated without salicylic acid application and irrigated with 100% ETr throughout the entire crop cycle (M1), as well as those subjected to water deficit during the flowering stage (M3) and the vegetative/flowering stages (M5), differed significantly from the other irrigation strategies. Application of 1.0 mM SAL increased APW in plants under water deficit during the flowering stage (M3) compared to those subjected to strategies M1, M2, M4, M5, and M6. Conversely, foliar application of 1.0 mM SAL increased APW under M2, M3, and M4 relative to plants exposed to the same water regime without SAL. No significant differences between SAL treatments (0 and 1 mM) were detected for the other irrigation strategies.

In the absence of salicylic acid, pulp yield (Figure 2e) of plants cultivated under full irrigation throughout the entire crop cycle (M1) was statistically higher than that of plants subjected to treatments M2, M3, M4, M5, and M6. In plants under deficit irrigation during the vegetative/flowering stages (M5), application 1.0 mM SAL resulted in higher PY values compared to those subjected to irrigation strategies M4 and M6. However, no significant differences in PY were observed among plants cultivated under M2, M3, and M5. When exploring the effects of salicylic acid application within each irrigation strategy, application of 1.0 mM SAL in plants subjected to irrigation strategies M3 and M5 resulted in higher PY values compared to those that did not receive this phytohormone. Conversely, under strategies M1 and M6, the absence of salicylic acid resulted in higher PY compared to plants cultivated with application of 1.0 mM. In contrast, no significant differences were observed between salicylic acid application in plants subjected to strategies M2 and M4.

The production stability index (Figure 2f) of sour passion fruit cultivated without salicylic acid application was not influenced by irrigation strategies. However, application of salicylic acid at a concentration of 1.0 mM resulted in the highest PSI in plants cultivated under water deficit during the flowering stage (M3), being statistically superior to those subjected to M1, M2, M4, M5, and M6. It was also observed that, at this SAL concentration, plants under full irrigation throughout the entire crop cycle (M1) achieved higher PSI compared to irrigation strategies M2, M4, M5, and M6. Salicylic acid application (0 and 1.0 mM) did not significantly affect PSI of sour passion fruit plants subjected to M2, M4, M5, and M6. Conversely, application of 1.0 mM SAL promoted increases of 30.00% and 89.39% in PSI for plants irrigated under M1 and M3, respectively, compared to those grown under the same conditions but without phytohormone application.

The interaction between deficit irrigation strategies and salicylic acid application significantly affected equatorial and polar fruit diameters, peel thickness, yield, and water use efficiency (Table 3S).

Regarding fruit equatorial diameter (Figure 3a), it was observed that in the absence of salicylic acid (0 mM), there were no significant differences among irrigation strategies, except for strategies M4 and M6. However, application of 1.0 mM SAL resulted in an increase in EDF in plants cultivated under 50% ETr during the flowering stage (M3), showing superiority compared to plants under strategy M1, M2, M4, M5, and M6. Salicylic acid at a concentration of 1.0 mM provided the highest EDF values when plants were cultivated under M3, M4, and M6 compared to fruits from plants grown under the same water conditions but without salicylic acid application (0 mM). For the remaining irrigation strategies (M1, M2, and M5), no differences were observed between the 0 and 1 mM salicylic acid concentrations.

**Figure 3:**
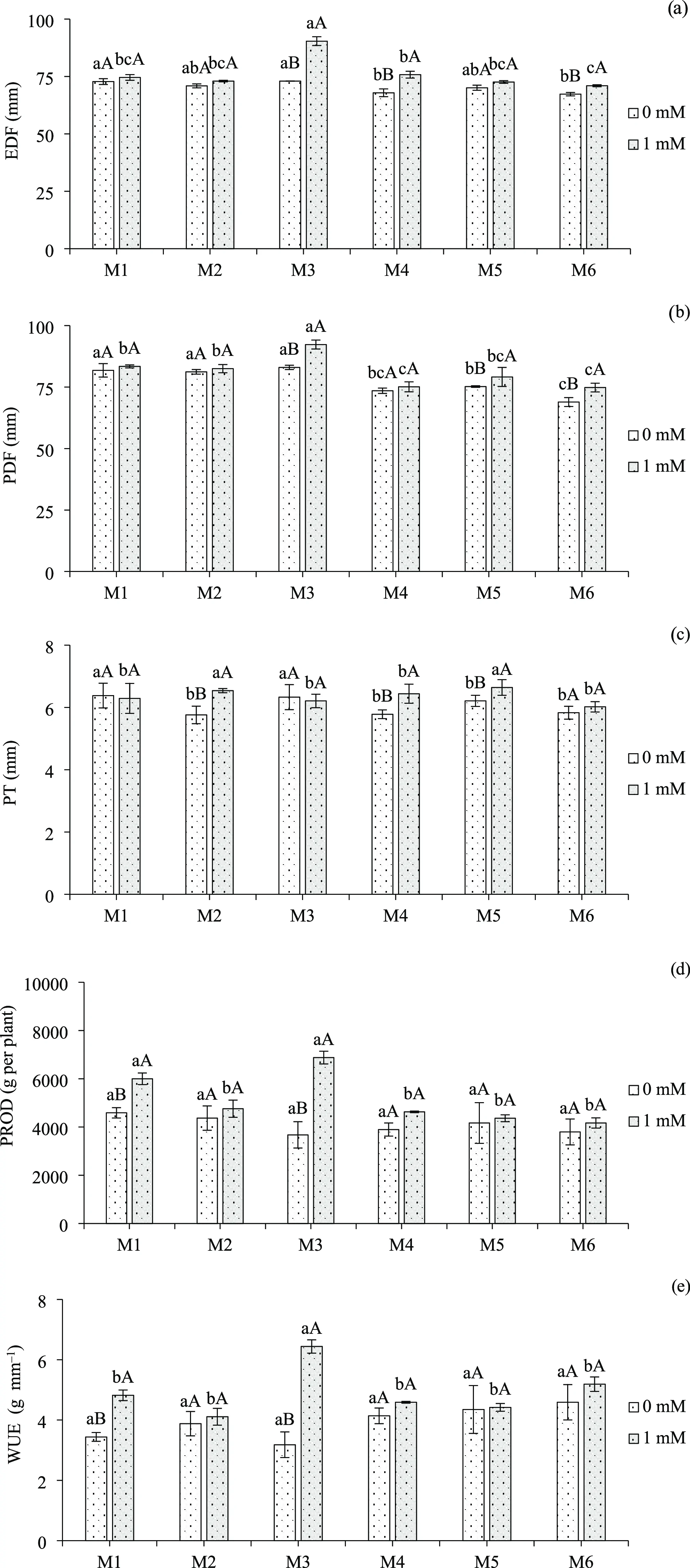
Equatorial diameter—EDF (a), polar diameter—PDF (b), peel thickness—PT (c), yield per plant—PROD (d), and water use efficiency—WUE (e) of sour passion fruit cv. Redondo Amarelo as a function of the interaction between deficit irrigation strategies and salicylic acid application. The same lowercase letters indicate no significant difference among deficit irrigation strategies (Scott–Knott, *p* ≤ 0.05), whereas the same uppercase letters indicate no significant difference between salicylic acid application (Tukey, *p* ≤ 0.05). Vertical bars represent the standard error of the mean (*n* = 4).

The polar diameter of fruits (Figure 3b) from sour passion fruit plants cultivated without salicylic acid application showed the highest values of 83.4, 82.5, and 92.3 mm under M1, M2, and M3, respectively, differing from of other irrigation strategies. In the presence of salicylic acid at a concentration of 1.0 mM, plants under deficit irrigation during the flowering stage (M3) exhibited higher PDF compared to those under M1, M2, M4, M5, and M6. In plants subjected to water deficit during the vegetative stage (M2), application of 1.0 mM SAL resulted in statistically higher PDF values compared to plants cultivated under M4, M5, and M6. When analyzing salicylic acid tested concentrations within each irrigation strategy, no significant differences were observed for plants subjected to M1, M2, and M4. Conversely, application of 1.0 mM SAL increased the polar diameter of sour passion fruit by 11.20%, 5.19%, and 8.56% under irrigation strategies M3, M5, and M6, respectively, compared to plants that did not receive this phytohormone (0 mM).

Regarding fruit peel thickness (Figure 3c), plants cultivated without salicylic acid application and irrigated with 100% ETr throughout the entire crop cycle (M1), as well as those subjected to water deficit during the flowering stage (M3), exhibited statistically higher PT values compared to plants under strategies M2, M4, M5, and M6. Application of 1.0 mM SAL resulted in higher PT values in plants cultivated under M2 and M5, differing significantly from those subjected to M1, M3, M4, and M6. Application of 1.0 mM SAL increased PT in plants cultivated under M2, M4, and M5, showing increases of 0.78, 0.66, and 0.43 mm, respectively, compared to plants grown under the same water conditions but without phytohormone application. For the remaining irrigation strategies, no significant differences were observed between plants treated with 0 and 1 mM salicylic acid.

Yield per plant (Figure 3d) of sour passion fruit cultivated in the absence of salicylic acid showed no significant differences among irrigation strategies. Conversely, when 1.0 mM salicylic acid was applied, the highest values of 5,998.92 and 6,877.65 g per plant were obtained under M1 and M3, respectively, differing significantly from plants irrigated under M2, M4, M5, and M6. When analyzing salicylic acid tested concentrations within each deficit irrigation strategy, application of 1.0 mM SAL increased yield in plants cultivated under M1 and M3, with values statistically higher than those of plants grown under the same water conditions but without phytohormone application. For the remaining irrigation strategies (M2, M4, M5, and M6), no significant differences were observed between plants treated with and without salicylic acid application.

Water use efficiency (Figure 3e) of plants cultivated without salicylic acid application did not show significant differences among irrigation strategies. However, application of 1.0 mM SAL resulted in the highest value of 6.44 g mm^–1^ in plants subjected to water deficit during the flowering stage (M3), being superior to those achieved under strategies M1, M2, M4, M5, and M6. Similar to what was observed for yield (Figure 3d), salicylic acid concentrations (0 and 1.0 mM) did not differ significantly under M2, M4, M5, and M6. Conversely, plants treated with 1.0 mM SAL and irrigated under M1 and M3 exhibited increases of 40.12% and 102.52%, respectively, compared to plants irrigated under the same water depths without salicylic acid application.

A significant effect was observed (Table 4S) for the interaction between deficit irrigation strategies (M) and salicylic acid application (SAL) on all postharvest variables analyzed.

The hydrogen potential (Figure 4a) of sour passion fruit pulp cultivated without salicylic acid reached the highest value under water deficit during the vegetative/flowering stages (M5), being higher than that observed under M6. However, no significant differences were observed in relation to plants subjected to M1, M2, M3, and M4. Under foliar application of 1.0 mM salicylic acid, plants irrigated under M4 achieved higher pH values compared to those subjected to M1, M5, and M6. When analyzing salicylic acid tested concentrations within each irrigation strategy, plants under M1, M3, M5, and M6 showed no significant differences between the 0 and 1.0 mM salicylic acid concentrations. However, application of 1.0 mM SAL increased pulp pH in plants irrigated with 50% ETr during the vegetative stage (M2) and fruiting stage (M4), respectively, compared to untreated plants under the same water conditions.

**Figure 4:**
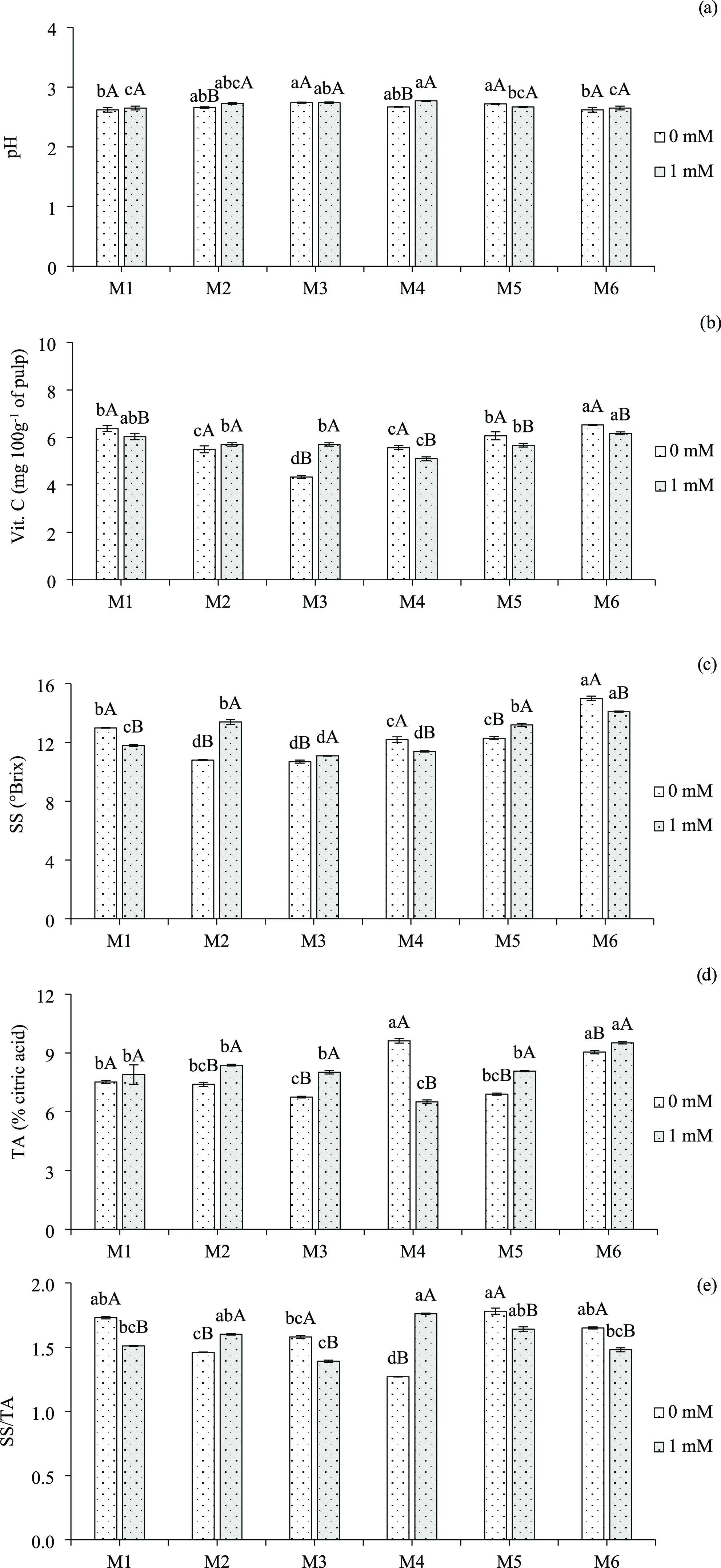
Hydrogen potential—pH (a), vitamin C content—Vit C. (b), soluble solids—SS (c), titratable acidity—TA (d), and the soluble solids to titratable acidity ratio—SS/TA (e) of sour passion fruit pulp cv. Redondo Amarelo as a function of the interaction between deficit irrigation strategies and salicylic acid application. Lowercase letters denote the absence of significant differences among deficit irrigation strategies according to the Scott–Knott test (*p* ≤ 0.05), whereas uppercase letters indicate the absence of significant differences between salicylic acid treatments based on Tukey’s test (*p* ≤ 0.05). Vertical bars correspond to the standard error of the mean (*n* = 4).

Regarding vitamin C content (Figure 4b) of sour passion fruit pulp, it was observed that, in the absence of salicylic acid, deficit irrigation during the flowering stage (M3) resulted in the minimum value of 4.33 mg 100 g^–1^ of pulp, differing significantly from plants cultivated under M1, M2, M4, M5, and M6. When analyzing salicylic acid tested concentrations within each irrigation strategy, application of 1.0 mM SAL increased vitamin C content in M3 compared to those that did not receive SAL (0 mM). Under irrigation strategies M1, M4, M5, and M6, application of 1.0 mM SAL resulted in lower vitamin C values than those obtained in the absence of this phytohormone. Under M2, no significant differences were observed between treatments with and without salicylic acid application.

For soluble solids (Figure 4c), water deficit during the vegetative/fruiting stage (M6) resulted in the highest value (15.00 °Brix) in the absence of salicylic acid. However, under this condition, no significant differences were observed between M3 and M4. In the presence of salicylic acid (1.0 mM), deficit irrigation during the vegetative/fruiting stages (M6) provided higher SS values than those achieved under M2, M3, M4, and M5. When analyzing the effect of 1.0 mM salicylic acid within each irrigation strategy, application of 1.0 mM SAL increased SS contents only under M2, M3, and M5, whereas reductions were observed under M1, M4, and M6 compared to plants cultivated under the same water conditions but treated with 0 mM SAL.

For titratable acidity (Figure 4d), it was observed that in the absence of salicylic acid, water deficit during M4 and M6 resulted in the highest values (9.62 and 9.05% citric acid, respectively), being higher than those found in plants cultivated under M1, M2, M3, and M5. It was also noted that no significant differences in TA were observed among plants irrigated under M1, M2, and M5 without salicylic acid application. In plants subjected to 50% ETr during the vegetative/fruiting stages (M6), application of salicylic acid at a concentration of 1.0 mM resulted in higher TA values (9.52% citric acid), differing from those receiving M1, M2, M3, M4, and M5. Salicylic acid application (0 and 1.0 mM) did not significantly influence TA of fruits produced under full irrigation (M1). However, application of 1.0 mM SAL reduced TA in plants irrigated under M4, while increasing titratable acidity in fruits from the other strategies (M2, M3, M5, and M6).

For the soluble solids to titratable acidity ratio (Figure 4e) of sour passion fruit, in the absence of salicylic acid application, the highest values of 1.73, 1.78, and 1.65 were obtained in plants subjected to strategies M1, M5, and M6, respectively. Application of salicylic acid at a concentration of 1.0 mM resulted in an increase in the SS/TA ratio in plants under M2 and M4. However, no significant differences were observed among M1, M2, M5, and M6 at the same salicylic acid concentration. In plants cultivated under M1, M3, M5, and M6, the absence of salicylic acid resulted in statistically higher SS/TA values compared to those obtained in plants that received 1.0 mM SAL. Conversely, application of this phytohormone increased the SS/TA ratio in plants subjected to M2 and M4.

There was an interaction between the factors (M × SAL) for reducing sugars, flavonoids, anthocyanins, and phenolic compounds in fruits of sour passion fruit cv. Redondo Amarelo (Table 5S).

Regarding reducing sugars (Figure 5a), it was observed that in the absence of salicylic acid application, the maximum value of 0.86 mg 100 g^–1^ of pulp was obtained in plants cultivated under deficit irrigation M6, being higher by 48.61%, 52.31%, 49.91%, 151.76%, and 7.94% compared to those subjected to treatments M1, M2, M3, M4, and M5, respectively. With salicylic acid application (1.0 mM), plants under M5 and M6 showed statistical superiority compared to those irrigated under strategies M1, M2, M3, and M4. Salicylic acid concentrations (0 and 1.0 mM) did not show significant differences in RS for plants subjected to M1, M2, and M3. Conversely, application of 1.0 mM SAL resulted in increases in RS when plants were irrigated with 50% ETr during the fruiting stage (M4), while it reduced RS under water deficit during M5 and M6 compared to plants cultivated without salicylic acid (0 mM).

**Figure 5:**
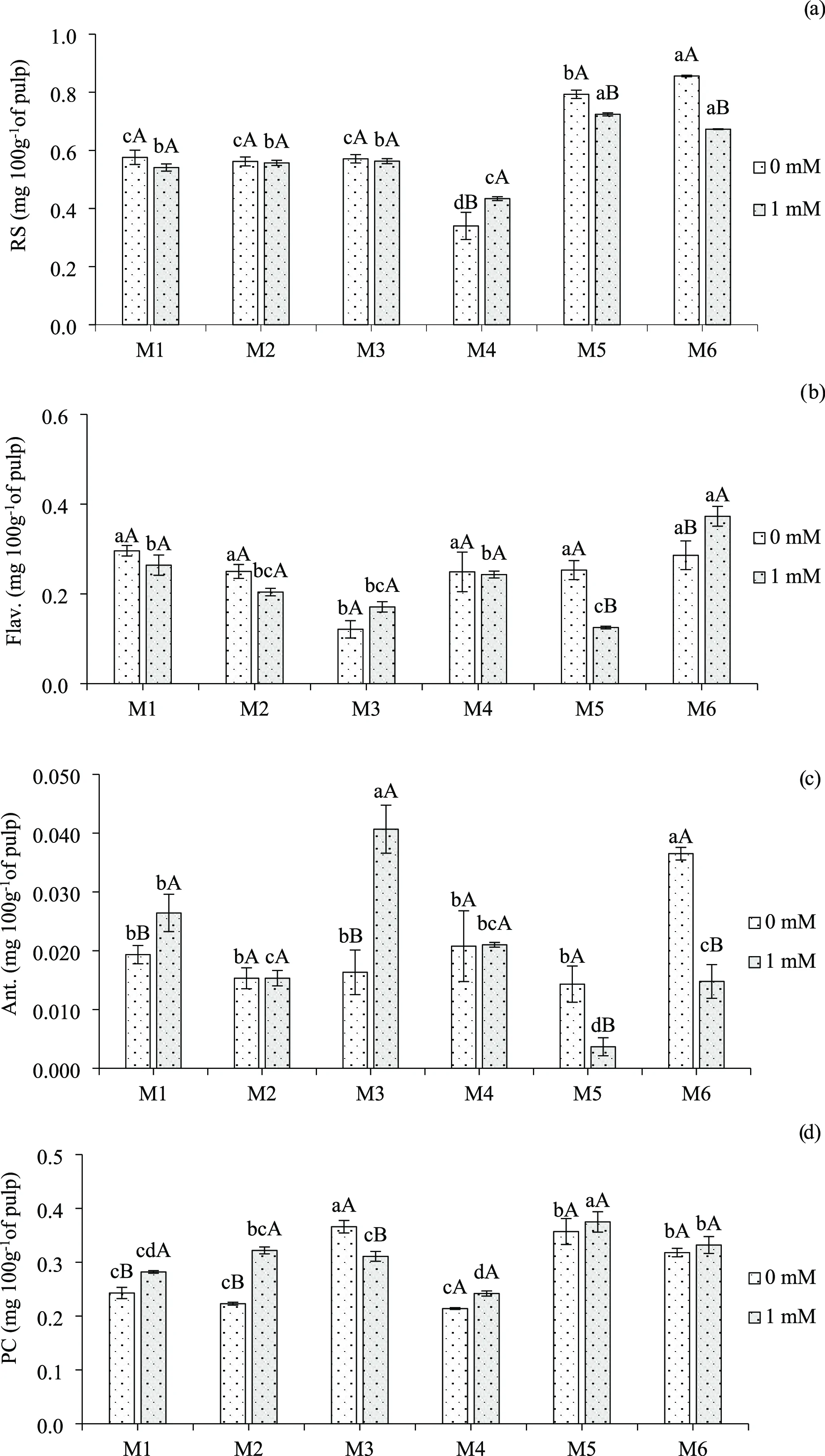
Reducing sugars—RS (a), flavonoids—Flav. (b), anthocyanins—Ant. (c), and phenolic compounds—PC (d) of sour passion fruit cv. Redondo Amarelo as a function of the interaction between deficit irrigation strategies and salicylic acid application. Lowercase letters denote the absence of significant differences among deficit irrigation strategies according to the Scott–Knott test (*p* ≤ 0.05), whereas uppercase letters indicate the absence of significant differences between salicylic acid treatments based on Tukey’s test (*p* ≤ 0.05). Vertical bars correspond to the standard error of the mean (*n* = 4).

For flavonoid content (Figure 5b), it was observed that in the absence of salicylic acid, plants irrigated with 50% ETr during the flowering stage (M3) exhibited the lowest value (0.12 mg 100 g^–1^ of pulp), differing significantly from those cultivated under M1, M2, M4, M5, and M6. With salicylic acid application, plants under water deficit during M6 showed statistical superiority compared to those irrigated under M1, M2, M3, M4, and M5. When analyzing salicylic acid tested concentrations within each irrigation strategy, significant differences were observed only for plants cultivated under deficit irrigation during the vegetative/flowering (M5) and vegetative/fruiting (M6) stages.

Similarly to what was observed for reducing sugars (Figure 5a), it was found that in the absence of salicylic acid, fruits of sour passion fruit plants showed increased anthocyanin contents (Figure 5c) under deficit irrigation during the vegetative/fruiting stages (M6), differing significantly from those subjected to M1, M2, M3, M4, and M5. Application of 1.0 mM salicylic acid increased anthocyanin contents in plants cultivated under water deficit during the flowering stage (M3) when compared to those irrigated with 100% ETr (M1). When analyzing the interaction between the factors (M × SAL), salicylic acid application promoted increases in anthocyanin contents in plants irrigated with 100% ETr throughout the entire crop cycle (M1) and under water deficit during the flowering stage (M3), respectively, compared to plants under the same water conditions but treated with 0 mM SAL. The lowest observed anthocyanin value (0.0037 mg 100 g^–1^ of pulp) occurred in plants subjected to salicylic acid application under deficit irrigation during the vegetative/flowering stages (M5).

Phenolic compounds (Figure 5d) were influenced by irrigation strategies in the absence of salicylic acid application, with M3 providing the highest value (0.37 mg 100 g^–1^ of pulp) compared to plants cultivated under M1, M2, M4, M5, and M6. When analyzing salicylic acid tested concentrations, application of 1.0 mM SAL increased phenolic compound contents in fruits of sour passion fruit plants irrigated under M1 and M2, while reducing those cultivated under water deficit during the flowering stage (M3). For the remaining irrigation strategies, no significant differences were observed between salicylic acid application (0 and 1.0 mM).

Changes in yield variables, postharvest fruit quality, and water use efficiency of sour passion fruit plants that were not subjected to salicylic acid application (Figure 6a) and those that received 1.0 mM SAL (b) can be observed in the Pearson correlation matrices (Figure 6b). For plants cultivated in the absence of salicylic acid (Figure 6a), a strong negative correlation was observed between the number of fruits per plant and average fruit weight (−0.95), average peel weight (−0.96), average pulp weight (−0.59), fruit equatorial diameter (−0.55), fruit polar diameter (−0.52), peel thickness (−0.52), hydrogen potential (−0.95), and phenolic compounds (−0.64). Conversely, positive correlations were observed among the remaining variables. Average fruit weight showed a strong positive correlation with pulp weight (0.79) and with EDF (0.71), PDF (0.65), and ST (0.64). Yield and water use efficiency showed a low degree of correlation with the evaluated variables. Water use efficiency was negatively correlated with pulp weight and fruit physical characteristics.

**Figure 6:**
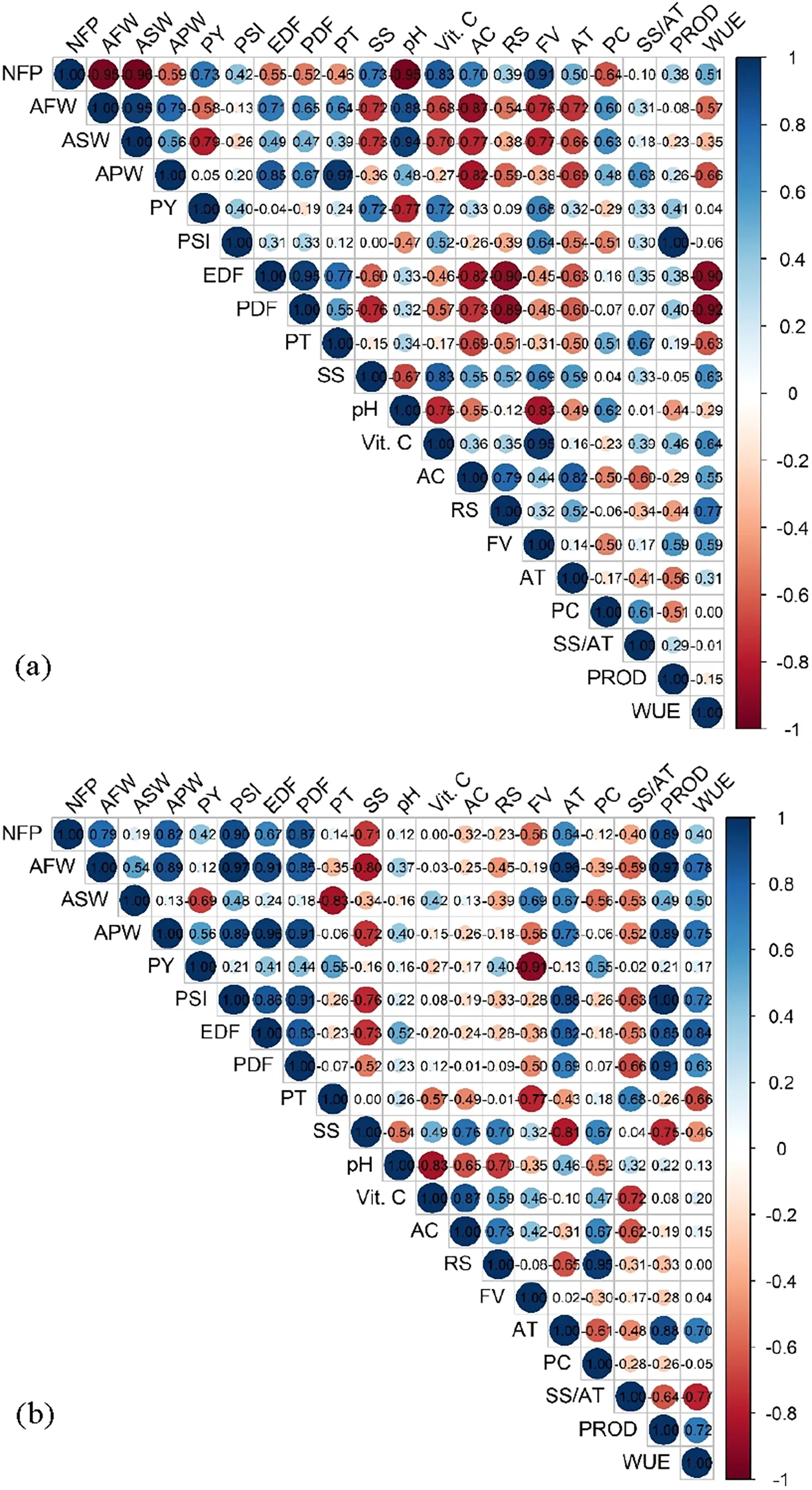
Pearson correlation matrices for the variables evaluated under irrigation strategies combined with 0 mM (a) or 1.0 mM (b) salicylic acid application.

Overall, yield variables were negatively correlated with postharvest variables, except for pH. Positive strong correlations (*r* ≥ 0.70) were observed only among soluble solids, vitamin C, titratable acidity, and reducing sugars. Vitamin C content showed a positive correlation with flavonoids (0.96), indicating a greater production of antioxidant compounds. Water use efficiency showed a positive correlation (*r* ≥ 0.50) with vitamin C, anthocyanins, reducing sugars, and flavonoids.

Conversely, when examining the correlation matrix for plants subjected to application of 1.0 mM salicylic acid (Figure 6b), a greater number of positive correlations was observed among yield variables. The number of fruits per plant (NFP) showed positive correlations (*r* ≥ 0.60) with average fruit weight (AFW), average pulp weight (APW), production stability index (PSI), equatorial fruit diameter (EDF), and polar fruit diameter (PDF). Average fruit weight and average peel weight exhibited strong positive correlations greater than 0.80 with PSI, EDF, and PDF. Peel weight showed a strong negative correlation only with pulp yield (PY) (−0.69). Yield and water use efficiency presented positive correlations (*r* ≥ 0.50) with all yield variables and fruit physical attributes, except for pulp yield, which showed a low correlation (0.17).

Overall, yield variables were positively correlated only with titratable acidity and negatively correlated with flavonoids, phenolic compounds, and the SS/TA ratio. For the remaining variables, correlations were lower than ±0.40. Vitamin C showed a strong positive correlation with titratable acidity (0.87), while exhibiting a strong negative correlation with the SS/TA ratio. Reducing sugars showed a strong positive correlation with phenolic compounds (0.95). Only anthocyanins showed a strong correlation with fruit yield (0.88) in sour passion fruit.

The principal component analysis (Figure 7) revealed the combined effects of deficit irrigation strategies (M) and salicylic acid application (SAL). The first two principal components accounted for 72% of the total variation, with PC1 explaining 46.1% and PC2 accounting for 25.9% of the variance. The first component showed positive correlations for AFW (0.92), APW (0.93), PSI (0.82), EDF (0.92), PDF (0.88), pH (0.64), and yield (PROD) (0.82), which were favored by groups 1 and 4. Still within PC1, negative correlations were observed for SS (−0.66) and RS (−0.66), which were favored only by group 3.

**Figure 7:**
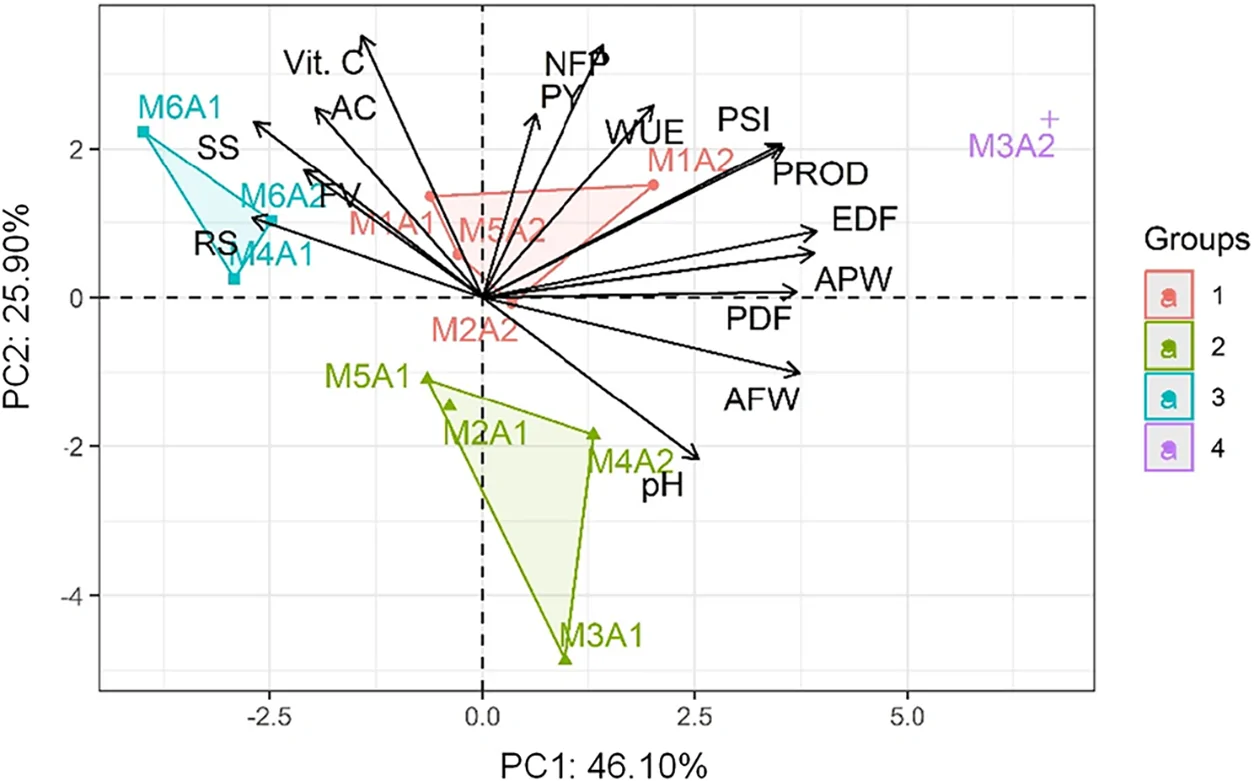
Two-dimensional representation of the principal component scores associated with deficit irrigation strategies (M) and salicylic acid application (SAL), together with the variables contributing to PC1 and PC2.

In the second component, positive correlations were observed only for NFP (0.86), vitamin C (0.73), and WUE (0.63), which clustered with groups 1 and 3. No negative correlations were observed in PC2.

## 4 Discussion

### 4.1 Effects of Water Deficit and Salicylic Acid Application on Yield Components

In the context of current climate change, several future scenarios project changes in rainfall regimes across different regions of the planet, with an increasing trend toward water scarcity, especially in semi–arid areas of Northeastern Brazil.^30^ According to Yuan et al.,^31^ the growing demand for water, combined with these climatic changes, is likely to further aggravate this situation, intensifying drought periods and their socioeconomic impacts on the agricultural sector.

However, the association between deficit irrigation strategies and the application of substances capable of inducing tolerance to such conditions is of great relevance. Salicylic acid (SAL) is a phenolic compound that modulates several physiological and biochemical pathways, acting primarily as a signaling molecule involved in plant defense and acclimation to abiotic stresses.^14,32^

In the present study, fruit number was negatively correlated with the other production variables of sour passion fruit (Figure 6a), suggesting that an increase in these traits was associated with a reduction in the number of fruits. This response may be related to the effects of deficit irrigation on leaf area development, which can impair carbon fixation, reduce photoassimilate production, and limit water uptake.^33^ Since fruit development depends on the accumulation of water and photoassimilates, it is strongly influenced by environmental and climatic conditions, which may affect fruit set, yield, and fruit quality.^34^

Changes in postharvest fruit quality induced by deficit irrigation at specific developmental stages may be associated with distinct physiological mechanisms, particularly during the cell division and cell expansion phases, which play a critical role in fruit production. Water deficit during these stages may limit carbon assimilation and reduce water availability within the tissues, thereby restricting fruit growth and development.^35^ This effect may be associated with the lower pulp yield (Figure 2e) observed in sour passion fruit, in which the control treatment showed superiority.

On the other hand, the negative effects of water deficit were mitigated for NFP (Figure 2a), AFW (Figure 2b), ASW (Figure 2c), and APW (Figure 2d) by salicylic acid application. According to Gao et al.,^36^ this phytohormone may act through multiple physiological pathways, enhancing drought tolerance by regulating stomatal aperture and maintaining osmotic homeostasis. These responses may improve nutrient uptake and stimulate the biosynthesis of antioxidant compounds, thereby maintaining the balance between the production and scavenging of reactive oxygen species (ROS).

An important point to highlight is that the behavior observed in the Pearson correlation for plants treated with 0 mM SAL (Figure 6a) was not observed with the application of 1.0 mM SAL (Figure 6a), in which a greater number of correlations related to yield variables was observed. This result is possibly associated with higher fruit production (Figure 2a), greater fruit weight (Figure 2b), higher pulp weight (Figure 2d), and larger equatorial (Figure 3a) and polar diameters (Figure 3b), which directly influenced the higher yield (Figure 3d) of sour passion fruit.

Similarly to what was observed for NFP (Figure 1A), a reduction in the production stability index (Figure 1F) was observed as a function of irrigation strategies. This reduction in PSI of sour passion fruit may be related to the negative effects of water deficit during the flowering stage, mainly due to alterations in the viability of the pollination process,^37^ which can ultimately lead to decreases in yield and, consequently, to greater production instability.

However, this effect can be mitigated by the application of 1.0 mM salicylic acid. Salicylic acid can act on several plant mechanisms, including seed germination, developmental progression, and floral induction,^38^ thereby favoring plant productivity. Furthermore, salicylic acid may promote cell division and cell expansion, thereby enhancing plant growth.^39^ Consequently, an increase in the photosynthetically active leaf area may improve light interception and CO_2_ assimilation, leading to greater photoassimilate production for fruit development. These physiological responses may explain the higher peel weight (Figure 2b), average pulp weight (Figure 2d), and fruit yield (Figure 3d) observed in sour passion fruit.

Regarding the physical characteristics of the fruits, Xue et al.^40^ reported that the fruit expansion stage is primarily regulated by cell expansion and is characterized by a high water demand. In this context, the reductions observed in equatorial diameter, polar diameter, and peel thickness in plants subjected to 50% ETr during the fruiting (M4), vegetative/flowering (M5), and vegetative/fruiting (M6) stages may be attributed to the greater sensitivity of these developmental stages to water deficit.

With salicylic acid application, increases in EDF and PDF were observed, especially in plants subjected to water deficit during the flowering stage (M3). According to Xin et al.,^41^ salicylic acid may increase chlorophyll content and enhance the activity of ribulose-1,5-bisphosphate carboxylase/oxygenase (RuBisCO), thereby improving photosynthetic efficiency. As a result, greater carbon assimilation and photoassimilate production may promote plant growth and fruit expansion, ultimately leading to the development of larger and more vigorous fruits.

Yield (Figure 3d) and water use efficiency (Figure 3e) were not individually influenced by irrigation strategies. Although deficit irrigation strategies did not differ significantly, this condition represents a common phenomenon under more severe climate change scenarios, which negatively impacts fruit yield and quality.^42^

However, increases were observed with the application of 1.0 mM salicylic acid under full irrigation throughout the entire crop cycle and when plants were subjected to 50% ETr during the flowering stage. Yield and water use efficiency showed stronger correlations in the presence of salicylic acid (Figure 6b), which may be associated with the beneficial effects of this phytohormone. This result is possibly related to greater efficiency in the redistribution of water and photoassimilates to the fruits due to increased CO_2_ assimilation rates, thereby promoting larger fruits (Figure 3A,3B) and fruits with greater filling (Figure 2c).

The negative effect of water deficit may be associated with an increase in the abortion of young organs, particularly reproductive structures,^43^ thereby affecting crop yield. On the other hand, with salicylic acid application, increases were observed in the evaluated variables, especially when associated with deficit irrigation during the flowering stage for yield and water use efficiency. Salicylic acid treatment may induce the expression of genes related to melatonin synthesis, increasing its endogenous levels,^44^ thereby improving fruit water retention capacity through the formation of a more cross–linked pectin network,^45^ ultimately resulting in higher crop yield and greater water use efficiency.

### 4.2 Effects of Water Deficit and Salicylic Acid Application on Postharvest Components

Irrigation exerts a significant impact on most postharvest fruit quality indicators, especially those related to the influence of water availability on the accumulation of photosynthetic products in plants.^46^ The accumulation of these compounds during ripening not only determines fruit sensory quality but also contributes to their stability.^47^ Salicylic acid, in turn, is widely recognized for increasing plant resilience to biotic and abiotic stresses.^48^ This compound can preserve the integrity of cell membranes, reduce respiration rate, and delay ripening, thereby maintaining fruit quality attributes.^49^

In the present study, the hydrogen potential (Figure 4a) of sour passion fruit did not show statistically significant differences among the different deficit irrigation strategies. However, application of 1.0 mM salicylic acid increased fruit pH, resulting in the highest values of 2.73 and 2.77 compared to fruits irrigated under the same conditions but without application of the compound. These values are consistent with Normative Instruction No. 37, of October 1, 2018, which establishes a minimum pH value of 2.70 for sour passion fruit.^26^

On the other hand, irrigation with 50% ETr during the vegetative/fruiting stages increased vitamin C content (Figure 4b) and soluble solids (Figure 4c), while reductions were observed for the other irrigation strategies. This result may be associated with the number of fruits (Figure 2a), since a higher fruit load can intensify competition for assimilates, further reducing the accumulation of soluble compounds such as SS,^50^ thus corroborating the hypothesis shown in Figure 6b, where a negative correlation (−0.71) between NFP and SS was observed. The beneficial effect of salicylic acid was observed for vitamin C in plants irrigated with 50% ETr during the flowering stage (M3), whereas for soluble solids, positive effects were observed under deficit irrigation during the vegetative stage (M2), flowering stage (M3), and the combined vegetative/flowering stages (M5). Soluble solids content (SS) is an indicator of sweetness and represents the main contribution to consumer acceptability,^51^ thus constituting a highly important chemical attribute of fruits for human consumption.

Deficit irrigation applied during the fruiting and vegetative/fruiting stages promoted an increase in titratable acidity (TA) of sour passion fruit (Figure 4d). This effect may be related to reduced fruit development, reflected in lower pulp weight (Figure 2d) and lower pulp yield (Figure 2e). Reduced tissue expansion and delayed physiological maturation may limit the dilution of accumulated organic acids, resulting in fruits with higher acidity. This response may occur because water restriction can reduce the translocation of photoassimilates and alter the balance between sugars and organic acids during fruit ripening.^52^

The increase in titratable acidity led to an increase in the SS/TA ratio (Figure 4e) in plants treated under M3. According to Li et al.,^52^ soluble solids (SS) and titratable acidity (TA) are important attributes affecting fruit quality, and their ratio is responsible for maintaining an appropriate sugar–acid balance, which determines market acceptance. In the presence of salicylic acid (1.0 mM), increases in TA of sour passion fruit were observed when plants were treated under M2, M3, M5, and M6.

Titratable acidity (TA) is directly related to the content of organic acids, which constitute the main respiratory substrates of fruits.^53^ During storage, a reduction in TA values is observed as a result of pyruvate decarboxylation occurring throughout the ripening process.^54^ However, the application of salicylic acid (SAL) may contribute to stabilizing titratable acid content. This effect may be attributed to the ability of salicylic acid to reduce respiration rates and inhibit ethylene biosynthesis, thereby delaying postharvest metabolism and preserving fruit quality.^55^

Cultivation under M6 resulted in an increase in reducing sugars (Figure 5a) in sour passion fruit. Salicylic acid application decreased reducing sugars (RS) in treatments M5 and M6, while increasing RS (Figure 5a) when plants were irrigated under M4. This increase under fruiting–stage water deficit may be associated with the effect of salicylic acid on enzymatic activities involved in sugar metabolism, particularly through inhibition of invertase, an enzyme that converts sucrose into glucose and fructose, thereby allowing sucrose levels to remain high.^56^ In addition, the accumulation of sugars and other solutes, combined with reduced water content, tends to decrease the osmotic potential of tissues, directly interfering with the biophysical growth of fruits,^57^ as evidenced by the production components observed in the present study.

Regarding flavonoid contents (Figure 5b), deficit irrigation during the flowering stage resulted in reductions of this variable. On the other hand, with salicylic acid application, increases in flavonoid contents were observed in plants irrigated under M3 and M6. This response may be associated with the effect of salicylic acid on inducing the synthesis of bioactive compounds, thereby enhancing the plant antioxidant system by stimulating the phenylpropanoid biosynthesis pathway.^58^ According to Helaly et al.,^59^ the antioxidant activities of salicylic acid play an important role in maintaining postharvest quality of crops.

Anthocyanin contents (Figure 5c) were reduced under treatments M1, M2, M3, M4, and M5, whereas an increase was observed when plants were cultivated under M6. Salicylic acid application was beneficial only under full irrigation throughout the entire crop cycle and under 50% ETr during the flowering stage. Salicylic acid application may delay fruit senescence by retarding ethylene production and inhibiting fruit respiration rate,^60^ in addition to promoting increased antioxidant activity.

Phenolic compounds (Figure 5d) were higher when plants were cultivated under M3. Conversely, a reduction in PC was observed under M3, while increases were recorded under M1 and M2. Water deficit is one of the main factors affecting plant production, mainly due to its impacts on photosynthesis, oxidative metabolism, and osmoregulation.^61^ Phenolic compounds exhibit antioxidant activity by scavenging free radicals and reducing oxidative damage to fruits, in addition to contributing to flavor and maintenance of fruit quality.^62^ Salicylic acid (SAL) application has been associated with the preservation of these compounds and the enhancement of antioxidant capacity, which may reduce oxidative damage, thereby extending fruit shelf life and delaying fruit senescence.^63^

Overall, improvements were observed in fruit yield, water use efficiency, and postharvest quality of sour passion fruit. Fruit quality is closely associated with the ripening and senescence processes, which involve weight loss, color changes, ethylene production, and respiration rate.^64^ Furthermore, salicylic acid application may delay fruit senescence by reducing ethylene production and inhibiting respiration rate, thereby improving postharvest quality.^65^

### 4.3 Synthesis of Physiological Responses and Comparison with Previous Studies

Table 1 summarizes studies investigating the effects of salicylic acid application in different plant species subjected to abiotic stress conditions or during postharvest storage. Collectively, these studies demonstrate that salicylic acid plays a key role in inducing stress tolerance mechanisms,^12^ contributing to the maintenance of photosynthetic activity,^41^ mitigation of oxidative damage,^12,66^ preservation of fruit quality,^45^ and enhancement of crop productivity.^14^

**Table 1:** Comparison of the Effects of Salicylic Acid in Different Plant Species under Abiotic Stress Conditions and during Postharvest Storage

reference	species	condition	key results
Present study	*Passiflora edulis* L.	Water deficit	Foliar application of 1.0 mM salicylic acid enhanced fruit yield, water use efficiency, and postharvest fruit quality, with the greatest benefits observed when water deficit occurred during the flowering stage.
Sobrinho et al.^14^	*Passiflora edulis* L.	Salt stress	Foliar application of salicylic acid at concentrations ranging from 1.0 to 1.4 mM partially mitigated the adverse effects of salinity stress, resulting in increased fruit yield and improved fruit quality of sour passion fruit.
Dantas et al.^68^	*Citrullus lanatus* L.	Water deficit	Foliar application of salicylic acid at concentrations ranging from 1.2 to 2.6 mM improved the physical characteristics and chemical composition of watermelon fruits irrigated with 60% of crop evapotranspiration replacement (ETr) during the flowering stage.
Acevedo et al.^12^	*Solanum tuberosum* L.	Water deficit	Exogenous foliar application of 1.0 mM salicylic acid alleviated the oxidative damage caused by water deficit by enhancing the accumulation of bioactive compounds and strengthening the antioxidant defense system.
Xin et al.^41^	*Zea mays* L.	Water deficit	Treatment with 5 mM salicylic acid increased chlorophyll and ribulose-1,5-bisphosphate carboxylase/oxygenase (RuBisCO) contents, thereby enhancing the photosynthetic capacity of the plants.
Batool et al.^45^	*Prunus armeniaca*	Storage	Application of 4 mM salicylic acid preserved total soluble solids, titratable acidity, color profile, ascorbic acid content, and total phenolic content during the postharvest storage of apricot fruits.
Baswal et al.^51^	*Citrus nobilis* L.	Storage	Application of 0.002 μmol L^–1^ salicylic acid preserved total soluble solids, titratable acidity, vitamin C content, and the sensory attributes of the fruit.
Xu et al.^66^	*Musa acuminata*	Storage	Maintenance of soluble sugar and total soluble solids contents, as well as antioxidant enzyme activity.

These beneficial effects may be primarily associated with enhanced antioxidant capacity,^12^ greater accumulation of bioactive compounds,^51^ and improved physiological performance under water deficit^44^ or salinity conditions.^14,67^ Furthermore, salicylic acid may contribute to preserving the physicochemical characteristics of fruits during storage by delaying deterioration and maintaining postharvest quality.^45,65^ Salicylic acid improves the physical and chemical attributes of fruits; however, its effectiveness largely depends on the phenological stage at which plants are exposed to water deficit. This response was demonstrated by Dantas et al.,^68^ who reported that foliar application of different salicylic acid concentrations in watermelon (*Citrullus lanatus* L.) produced distinct responses according to the phenological stage during which water deficit occurred, indicating that the timing of stress is a key factor determining the effectiveness of salicylic acid.

The findings of the present study support previous evidence by demonstrating that foliar application of 1.0 mM salicylic acid improved fruit yield, water use efficiency, and the physicochemical attributes of sour passion fruit. However, the response to salicylic acid was not consistent across all deficit irrigation strategies. The beneficial effects were most pronounced when water deficit was imposed during the flowering stage, whereas less expressive or inconsistent responses were observed during the other phenological stages. These findings suggest that the effectiveness of salicylic acid may depend not only on the plant species and the intensity of abiotic stress but also on the timing of water deficit during crop development.

Although the potential of salicylic acid to mitigate abiotic stress has been widely demonstrated in several crop species, studies involving sour passion fruit under semiarid conditions remain limited. Therefore, the present study contributes to advancing current knowledge by identifying the flowering stage as the phenological phase in which water deficit most strongly influences the effectiveness of salicylic acid, providing a basis for optimizing its use as a complementary management strategy under water-limited conditions.

## 5 Conclusions

Deficit irrigation affected the physical and chemical attributes, as well as the yield components of sour passion fruit, differently according to the phenological stage. The greatest reductions in yield and fruit quality were observed when water deficit occurred during the fruiting and vegetative/fruiting stages, indicating that these developmental periods are the most sensitive to water restriction. In contrast, deficit irrigation imposed during the flowering stage did not compromise crop performance, particularly when combined with foliar application of 1.0 mM salicylic acid.

The beneficial effects of salicylic acid varied according to the irrigation strategy. Foliar application of 1.0 mM salicylic acid enhanced tolerance to water deficit during the flowering stage by increasing fruit number, pulp weight, production stability index, and water use efficiency, while also improving postharvest quality attributes, including pH, vitamin C, soluble solids, titratable acidity, the soluble solids/titratable acidity ratio, reducing sugars, flavonoids, anthocyanins, and phenolic compounds. However, under the other deficit irrigation strategies, the beneficial effects of salicylic acid were limited or inconsistent, suggesting that its effectiveness, as well as the optimal concentration, depends on the timing of water restriction during crop development.

Based on these findings, foliar application of 1.0 mM salicylic acid can be recommended as a complementary management strategy for sour passion fruit cultivated under moderate water deficit during the flowering stage, where it improved both yield and postharvest fruit quality. Future studies should investigate the physiological, biochemical, and molecular mechanisms underlying these responses and evaluate additional salicylic acid concentrations in combination with alternative deficit irrigation strategies to optimize its use under agricultural conditions.

## Supplementary Material



## Data Availability

The data underlying the findings of this study are included in the article. Additional information may be obtained from the corresponding author upon request.

## Notes

Water consumption of yellow round passion fruit under different irrigation strategies, estimated through drainage lysimetry (Table 1S); summary of the analysis of variance for number of fruits per plant (NFP), average fruit weight (AFW), peel weight (ASW), pulp weight (APW), pulp yield (PY), and production stability index (PSI) (Table 2S); summary of the analysis of variance for equatorial diameter (EDF), polar diameter (PDF), peel thickness (PT), yield (PROD), and water use efficiency (WUE) (Table 3S); summary of the analysis of variance for hydrogen potential (pH), vitamin C content (Vit. C), soluble solids (SS), titratable acidity (TA), and the soluble solids to titratable acidity ratio (SS/TA) (Table 4S); summary of the analysis of variance for reducing sugars (RS), flavonoids (FV), anthocyanins (ANT), and phenolic compounds (PC) of sour passion fruit cv. Redondo Amarelo subjected to deficit irrigation strategies and salicylic acid (Table 5S) (PDF)
