# Supplementary material for: Physical and Chemical Attributes and Yield of Sour Passion Fruit Grown under Deficit Irrigation Strategies and Salicylic Acid Application
Source: ACS Omega. 2026 Sep 23;11(39):59168–83. doi: 10.1021/acsomega.6c04904 (PMC13648103; doi:10.1021/acsomega.6c04904)
Supplement: Supplementary file 1 [file ao6c04904_si_001.pdf]

Physical and Chemical Attributes and Yield of Sour Passion Fruit Grown under Deficit Irrigation Strategies and Salicylic Acid Application

Allesson R. de Souza[1], Geovani S. de Lima[2], André A. R. Da Silva[3], Carlos Alberto V. de Azevedo[1], Lucyelly Dâmela A. Borborema[1], Kheila G. Nunes[1], Denis S. Costa[1], Larissa Fernanda S. Santos[1], Brencarla de M. Lima[4], Valeska Karolini N. Oliveira[1], Smyth T. de A. Silva[1], Vitor Manoel B. da Silva[1], Edilene D. de Araújo[5], Allysson Jonhnny T. Mendonça[1], Lauriane A. dos A. Soares[2], Hans R. Gheyi[1]*

1 Academic Unit of Agricultural Engineering, Federal University of Campina Grande, Campina Grande 58429-900, Brazil:
2 Academic Unit of Agrarian Sciences, Federal University of Campina Grande, Pombal 58840-000, Brazil
3 Academic Unit of Agronomy, Universidade Federal do Oeste do Pará, Juruti 68170-000, Brazil
4 Food Technology Department, State University of Campinas, Campinas 13083-862, SP, Brazil
5 Postgraduate Program in Engineering and Management of Natural Resources, Federal University of Campina Grande 58430-380, Brazil:
* Correspondence: allesson.ramos@estudante.ufcg.edu.br; Tel.: +55(83) 99387-0752

LIST OF CONTENT SUPPLEMENT

Table 1S. Water consumption of yellow round passion fruit under different irrigation strategies, estimated through drainage lysimetry. 3

Table 2S. Summary of the analysis of variance for number of fruits per plant (NFP), average fruit weight (AFW), peel weight (ASW), pulp weight (APW), pulp yield (PY), and production stability index (PSI) of sour passion fruit cv. Redondo Amarelo subjected to deficit irrigation strategies and salicylic acid. 4

Table 3S. Summary of the analysis of variance for equatorial diameter (EDF), polar diameter (PDF), peel thickness (PT), yield (PROD), and water use efficiency (WUE) of sour passion fruit cv. Redondo Amarelo subjected to deficit irrigation strategies and salicylic acid. 5

Table 4S. Summary of the analysis of variance for hydrogen potential (pH), vitamin C content (Vit. C), soluble solids (SS), titratable acidity (TA), and the soluble solids to titratable acidity ratio (SS/TA) of sour passion fruit cv. Redondo Amarelo subjected to deficit irrigation strategies and salicylic acid. 6

Table 5S. Summary of the analysis of variance for reducing sugars (RS), flavonoids (FV), anthocyanins (ANT), and phenolic compounds (PC) of sour passion fruit cv. Redondo Amarelo subjected to deficit irrigation strategies and salicylic acid. 7

Table 1S. Water consumption of yellow round passion fruit under different irrigation strategies, estimated through drainage lysimetry.

Irrigation strategy	DAT	Water consumption (mm)	
		A1	A2
Full irrigation – M1	30 – 196	1483.55	1384.03
Vegetative – M2	30 – 90	1251.04	1285.45
Flowering – M3	90 – 136	1284.01	1185.86
Fruiting – M4	136 – 196	1046.26	1119.16
Vegetative + Flowering – M5	30 – 136	1063.57	1097.71
Vegetative + Fruiting – M6	30 – 90 /136 – 196	918.28	892.36

The area of the pot used was 0.180 m^2.

Table 2S. Summary of the analysis of variance for number of fruits per plant (NFP), average fruit weight (AFW), peel weight (ASW), pulp weight (APW), pulp yield (PY), and production stability index (PSI) of sour passion fruit cv. Redondo Amarelo subjected to deficit irrigation strategies and salicylic acid.

Source of variation	DF	Mean squares					
		NFP	AFW	ASW	APW	PY	PSI
Irrigation strategies (M)	5	44.30^{ns}	4691.19^{***}	503.81^{***}	2121.25^{***}	$7.94\text{x}10^{-3ns}$	0.11^{***}
Salicylic acid (SAL)	1	133.33^{*}	3313.45^{**}	40.54^{ns}	2423.72^{***}	$6.05\text{x}10^{-3ns}$	0.61^{**}
Interaction (M × SAL)	5	162.68^{**}	1598.67^{***}	1771.51^{***}	500.26^{***}	$3.53\text{x}10^{-2**}$	0.13^{**}
Blocks	3	61.14^{ns}	33.45^{ns}	24.16^{ns}	1.07^{ns}	$4.54\text{x}10^{-3ns}$	0.05^{ns}
Residue	33	27.01	161.33	38.17	37.71	$4.24\text{x}10^{-3}$	0.025
CV (%)		14.00	8.61	6.36	7.21	11.01	15.82

DF, degrees of freedom; CV, coefficient of variation. Asterisks indicate statistical significance at $p \leq 0.05$ (*), $p \leq 0.01$ (**), and $p \leq 0.001$ (***), whereas ^{ns} indicates a non-significant effect ($p > 0.05$).

Table 3S. Summary of the analysis of variance for equatorial diameter (EDF), polar diameter (PDF), peel thickness (PT), yield (PROD), and water use efficiency (WUE) of sour passion fruit cv. Redondo Amarelo subjected to deficit irrigation strategies and salicylic acid.

Source of variation	DF	Mean squares				
		EDF	PDF	PT	PROD	WUE
Irrigation strategies (M)	5	152.56***	276.33***	0.25**	3.05x10^{5*}	1.03^{ns}
Salicylic acid (SAL)	1	416.32***	184.97***	1.14**	1.63x10^{7***}	11.98***
Interaction (M × SAL)	5	74.20***	20.25*	0.29**	3.2x10^{6**}	2.87**
Blocks	3	2.40^{ns}	4.37^{ns}	0.010^{ns}	1.09x10^{5ns}	0.84^{ns}
Residue	33	4.44	5.87	0.042	6.62x10^{4}	0.51
CV (%)		2.88	3.06	3.68	15.46	16.24

DF, degrees of freedom; CV, coefficient of variation. Asterisks indicate statistical significance at $p \leq 0.05$ (*), $p \leq 0.01$ (**), and $p \leq 0.001$ (***), whereas ^{ns} indicates a non-significant effect ($p > 0.05$).

Table 4S. Summary of the analysis of variance for hydrogen potential (pH), vitamin C content (Vit. C), soluble solids (SS), titratable acidity (TA), and the soluble solids to titratable acidity ratio (SS/TA) of sour passion fruit cv. Redondo Amarelo subjected to deficit irrigation strategies and salicylic acid.

Source of variation	DF	Mean squares				
		pH	Vit. C	SS	TA	SS/TA
Irrigation strategies (M)	5	$1.39 \times 10^{-2**}$	$2.09 \times 10^{-2***}$	11.73^{***}	3.81^{***}	0.055^{***}
Salicylic acid (SAL)	1	$1.23 \times 10^{-2**}$	1.48×10^{-4ns}	0.42^{*}	0.44^{*}	0.0034^{ns}
Interaction (M × SAL)	5	$5.1 \times 10^{-4*}$	$1.01 \times 10^{-2***}$	4.32^{***}	5.52^{***}	0.16^{***}
Blocks	3	5.71×10^{-4ns}	4.81×10^{-2ns}	0.052^{ns}	0.063^{ns}	0.05^{ns}
Residue	33	1.16×10^{-3}	4.05×10^{-2}	0.04	0.11	0.02
CV (%)		1.49	3.52	1.73	4.06	4.83

DF, degrees of freedom; CV, coefficient of variation. Asterisks indicate statistical significance at $p \leq 0.05$ (*), $p \leq 0.01$ (**), and $p \leq 0.001$ (***), whereas ^{ns} indicates a non-significant effect ($p > 0.05$).

Table 5S. Summary of the analysis of variance for reducing sugars (RS), flavonoids (FV), anthocyanins (ANT), and phenolic compounds (PC) of sour passion fruit cv. Redondo Amarelo subjected to deficit irrigation strategies and salicylic acid.

Source of variation	DF	Mean squares			
		RS	FV	ANT	PC
Irrigation strategies (M)	5	$1.39 \times 10^{-2*}$	$3.39 \times 10^{-3***}$	$4.47 \times 10^{-4***}$	$3.39 \times 10^{-3***}$
Salicylic acid (SAL)	1	$1.23 \times 10^{-2**}$	1.85×10^{-4ns}	1.78×10^{-4ns}	1.85×10^{-4ns}
Interaction (M × SAL)	5	$5.1 \times 10^{-4**}$	$1.11 \times 10^{-3**}$	$5.63 \times 10^{-4**}$	$1.11 \times 10^{-3**}$
Blocks	3	5.71×10^{-4ns}	5.71×10^{-4ns}	2.33×10^{-4ns}	5.71×10^{-4ns}
Residue	33	1.16×10^{-3}	1.95×10^{-3}	1.56×10^{-5}	1.95×10^{-3}
CV (%)		1.49	8.61	15.21	7.05

DF, degrees of freedom; CV, coefficient of variation. Asterisks indicate statistical significance at $p \leq 0.05$ (*), $p \leq 0.01$ (**), and $p \leq 0.001$ (***), whereas ^{ns} indicates a non-significant effect ($p > 0.05$).
